# Effects of fatty acid profile of supplements on intake, performance, carcass traits, meat characteristics, and meat sensorial analysis of feedlot *Bos indicus* bulls offered a high-concentrate diet

**DOI:** 10.1093/tas/txaa142

**Published:** 2020-08-05

**Authors:** Carolina Costa, Ramon Rizzieri, Gabriel Melo, Leonardo Müller, Daniela Estevan, Rodrigo Pacheco, Danilo Millen, Angélica Pereira, Mariana Zanatta, Bruno Cappellozza, Rafael Cervieri, Cyntia Martins, Mário Arrigoni

**Affiliations:** 1 Faculdade de Medicina Veterinária e Zootecnia, Universidade Estadual Paulista, Botucatu, SP, Brazil; 2 EMPAER, Cuiabá, MT, Brazil; 3 Faculdade de Ciências Agrárias e Tecnológicas, Universidade Estadual Paulista, Dracena, SP, Brazil; 4 Faculdade de Medicina Veterinaria e Zootecnia, Universidade de São Paulo, Pirassununga, SP, Brazil; 5 Nutricorp, Araras, SP, Brazil; 6 Nutribeef Consultoria, Botucatu, SP, Brazil

**Keywords:** *Bos indicus*, fatty acid profile, lipid sources, performance, sensorial analysis

## Abstract

This experiment was designed to evaluate the effects of lipid source and fatty acid (FA) profile on intake, performance, carcass characteristics, expression of enzymes, and sensorial analysis of *Bos indicus* animals offered a high-concentrate diet. On day 0, 96 noncastrated animals were blocked by initial body weight (400 ± 19.3 kg), randomly allocated to 1 of 24 pens (4 animals/pen), and pens were randomly assigned to receive: 1) control: basal diet composed of whole cottonseed and corn germ as lipid substrates (CONT; *n* = 6), 2) calcium salts of fatty acids (CSFA) of soybean: CSFA of soybean oil as replacement for whole cottonseed and corn germ (calcium salts of soybean oil [CSSO]; *n* = 6), 3) CSFA-Blend: CSFA of palm, cottonseed, and soybean oil as replacement for whole cottonseed and corn germ (calcium salts of vegetable oils [CSVO]; *n* = 6), and 4) Mix: basal diet containing whole cottonseed, corn germ, and CSVO (MIXT; *n* = 6). Experiment lasted 108 d and performance, ultrasound measurements, as well as carcass characteristics were evaluated. Additionally, meat FA profile, expression of enzymes involved in lipid metabolism, and sensorial analysis were evaluated. No treatment effects were observed on performance variables, ultrasound, and carcass traits (*P* ≥ 0.22), whereas animals receiving CONT had a greater intake of C10:0, C16:0, C16:1 *trans*-9, C18:1 *cis*-9, C18:2, C18:3, total FA, monounsatured FA (MUFA), and polyunsaturated FA (PUFA) vs. CSSO and MIXT (*P* < 0.05). Conversely, intake ratios of saturated FA (SFA):MUFA and SFA:PUFA were all reduced for CONT vs. other treatments. Meat obtained from CONT animals had greater colorimetric (L*, a*, and b*) values vs. MIXT (*P* < 0.01). On meat FA profile, CONT increased C18:0 vs. supplementation with calcium salts (*P* < 0.02) and supplementation with CSSO yielded greater meat concentrations of C18:1 *trans*-10 and C18:2 CLA intermediates (*P* < 0.01). Expression of SREBP-1, SCD, and LPL was downregulated for CSSO (*P* < 0.05). For sensorial analysis, regular flavor was greater (*P* = 0.01) for CSSO vs. other treatments, but also greater aroma (*P* = 0.05) vs. CONT and CSVO. In summary, addition of different lipid sources with varying FA profiles into high-concentrate diets did not affect performance and carcass characteristics of *B. indicus* animals, but supplementation with calcium salts of soybean oil inhibited the mRNA expression of enzymes involved in lipid metabolism, whereas flavor and aroma were positively affected by this lipid source.

## INTRODUCTION

Over the last 10 yr, ether extract (EE) content of feedlot diets has been increasing in United States and Brazil (Pinto and Millen, 2016; Samuelson et al., 2016), whereas the major goals of this strategy are to increase dietary energy density and to improve feed efficiency of the herd (NASEM, 2016; Hales et al., 2017). More specifically in Brazil, the primary lipid sources offered to feedlot cattle are whole cottonseed and calcium salts of fatty acids (CSFA; Pinto and Millen, 2016). These sources differ in fatty acid (FA) profile and biohydrogenation rate, which ultimately result in a release of FA in the rumen and depending on its saturation profile, might affect rumen bacteria at a greater or lower extent (Jenkins, 1993). Additionally, rumen biohydrogenation significantly alters the FA profile absorbed in the small intestine and, consequently, the FA profile of milk and meat is different from what was offered to the herd through the diet (Jenkins et al., 2008).

Previous studies demonstrated that not only the source, but also the FA profile of the supplement, affects productive responses of ruminants. In grazing dairy cattle, de Souza et al. (2017) demonstrated that CSFA sources containing greater amounts of palmitic and oleic FA yielded greater productive responses vs. a linoleic-based source. In beef cattle, Fiorentini et al. (2013, 2014) reported that source and FA profile of the CSFA supplement affected nutrient intake and digestibility of beef animals offered a 60% roughage diet. Moreover, it is well-reported that FA might affect the expression of genes and enzymes involved in lipid metabolism in several tissues, such as meat (Choi et al., 2013), as well as meat acceptability, such as flavor and taste (Smith et al., 2006). Based on this rationale, we hypothesized that the FA profile of the supplement and diet would affect intake, performance, carcass traits, meat FA profile, and sensorial analysis of *Bos indicus* animals offered a high-concentrate diet. Therefore, our objective was to evaluate the effects of supplement and diet FA profile on intake, performance, carcass traits, meat FA profile, and sensorial analysis of *B. indicus* animals offered a high-concentrate diet.

## MATERIALS AND METHODS

This experiment was conducted at the experimental feedlot from the Departamento de Melhoramento e Nutrição Animal, located in Botucatu, São Paulo, Brazil (22°53’25” S, 48°27’19” W, and elevation of 828 m) from August to November 2016. All animals utilized herein were cared for in accordance with acceptable practices and experimental protocols reviewed and approved by the FMVZ/UNESP Institutional Animal Care and Use Committee (IACUC #117/2015).

### Animals, Diets, and Housing

The experimental design used herein was randomized complete block design (RCBD). On day 0 of the experiment, 96 noncastrated Nellore animals were blocked by initial shrunk body weight (BW = 400 ± 19.3 kg; initial age = 22 ± 3 mo) and randomly allocated to 1 of 24 pens (4 animals/pen). Within blocks (*n* = 6), pens were randomly assigned to 1 of 4 treatments: 1) control: basal diet composed of whole cottonseed and corn germ as lipid substrates (CONT; *n* = 6), 2) CSFA-Soybean: basal diet containing CSFA of soybean oil as replacement for whole cottonseed and corn germ (CSSO; Nutri Gordura, Nutricorp, Araras, SP, Brazil; *n* = 6), 3) CSFA-Blend: basal diet containing CSFA of palm, cottonseed, and soybean oil as replacement for whole cottonseed and corn germ (calcium salts of vegetable oils [CSVO]; Nutri Gordura Terminação, Nutricorp, Araras, SP, Brazil; *n* = 6), and 4) Mix: basal diet containing whole cottonseed, corn germ, and CSVO (MIXT; *n* = 6). A complete description of the FA profile of the lipid feedstuffs is reported in Table 1, meanwhile Tables 2 and 3 report the feedstuff composition and nutrient profile of the diets offered during the adaptation, growing, and finishing periods, respectively.

**Table 1.** FA profile of the lipid feedstuffs used in the present experiment

Fatty acid, % total FA	WCS	CGN	CSSO	CSVO
Capric acid (C10:0)	0.00	0.00	0.03	0.12
Lauric acid (C12:0)	0.39	0.00	0.33	0.94
Myristic acid (C14:0)	0.60	0.04	0.72	0.92
Pentadecanoic acid (C15:0)	0.06	0.01	0.13	0.09
Palmitic acid (C16:0)	14.54	11.8	18.48	29.65
Palmitoleic acid (C16:1)	0.51	0.13	0.59	0.21
Margaric acid (C17:0)	0.19	0.08	0.30	0.16
Stearic acid (C18:0)	3.39	2.65	6.95	5.45
Elaidic acid (C18:1)	0.00	0.00	4.95	4.08
Oleic acid (C18:1)	42.51	33.08	25.65	31.93
Linoleic acid (C18:2)	31.54	48.20	37.00	23.53
Linolenic acid (C18:3)	3.79	1.00	3.21	1.73
Arachidic acid (C20:0)	0.75	0.59	0.48	0.35
Eicosenoic acid (C20:1)	0.63	0.23	0.20	0.12
Behenic acid (C22:0)	0.57	0.18	0.52	0.37
Lignoceric acid (C24:0)	0.33	0.31	0.26	0.20
Unknown FA	0.20	1.70	0.20	0.15
SFA	20.82	15.66	28.40	38.25
Monounsaturated FA (MUFA)	43.65	33.44	31.39	36.34
PUFA	35.33	49.20	40.21	25.26

WCS = whole cottonseed; CGN = corn germ.

**Table 2.** Composition of the diets offered during adaptation (ADP), growing (GRO), and finishing (FIN) phases of the present experiment

Item, % dry matter	ADP				GRO				FIN			
	CONT	CSSO	CSVO	MIXT	CONT	CSSO	CSVO	MIXT	CONT	CSSO	CSVO	MIXT
Sugarcane bagasse	23.73	23.73	23.73	23.73	20.90	20.90	20.90	20.90	11.69	11.88	11.88	11.88
Ground corn	26.55	27.46	27.46	27.46	22.60	28.59	28.59	25.42	37.83	60.88	60.88	51.81
Citrus pulp	21.24	25.99	25.99	23.16	20.34	27.12	27.12	24.01	14.92	10.65	10.65	13.05
Peanut meal	15.82	18.08	18.08	16.95	12.99	17.40	17.40	15.25	2.38	8.94	8.94	4.75
Whole cottonseed	4.52	–	–	2.26	9.04	–	–	4.52	13.31	–	–	9.51
Corn germ	4.52	–	–	2.26	10.51	–	–	5.08	16.16	–	–	3.33
Nutri Gordura	–	1.13	–	–	–	2.37	–	–	–	3.94	–	–
Nutri Gordura Terminação	–	–	1.13	0.56	–	–	2.37	1.19	–	–	3.94	1.97
Mineral-vitamin mix^*a*^	3.50	3.50	3.50	3.50	3.50	3.50	3.50	3.50	3.52	3.52	3.52	3.52
Water	0.11	0.11	0.11	0.11	0.11	0.11	0.11	0.11	0.19	0.19	0.19	0.19

ADP = adaptation diet offered from days 0 to 10 of the experiment; GRO = growing diet offered from days 11 to 35 of the experiment; FIN = finishing diet offered from days 36 to 108 of the experiment.

^*a*^Mineral-vitamin mix contained 15.2% Ca, 2.6% S, 1.5% Mg, 4.2% Na, 2.0% P, 1,350 ppm Zn, 148 ppm Mn, 380 ppm Cu, 10.8 ppm I, 5 ppm Se, 43.2 ppm Co, 32.3% urea, and 720 ppm sodium monensin.

**Table 3.** Nutrient profile of the diets offered during adaptation (ADP), growing (GRO), and finishing (FIN) phases of the present experiment

Nutrient profile^*a*^	ADP				GRO				FIN			
	CONT	CSSO	CSVO	MIXT	CONT	CSSO	CSVO	MIXT	CONT	CSSO	CSVO	MIXT
DM	65.0	65.0	65.0	65.0	67.0	66.0	66.0	66.0	68.0	68.0	68.0	68.0
NDF, % DM	34.9	34.3	34.3	34.5	33.8	32.3	32.3	33.1	26.5	22.3	22.3	25.2
peNDF, % DM	21.0	20.0	20.0	21.0	21.0	18.0	18.0	20.0	16.0	11.0	11.0	15.0
CP, % DM	16.4	16.4	16.4	16.4	16.2	16.2	16.2	16.2	13.5	13.5	13.5	13.5
EE, % DM	3.1	3.1	3.1	3.1	4.0	4.0	4.0	4.0	5.5	5.5	5.5	5.5
Starch, % DM	21.6	21.5	21.5	21.9	19.8	22.3	22.3	20.9	31.2	44.7	44.7	38.8
Ca, % DM	1.13	1.36	1.36	1.24	1.11	1.52	1.52	1.32	0.97	1.36	1.36	1.17
P, % DM	0.35	0.34	0.34	0.35	0.35	0.34	0.34	0.35	0.35	0.35	0.35	0.35
TDN, % DM	72.0	72.0	72.0	72.0	73.0	74.0	74.0	74.0	78.0	81.0	81.0	79.0
DE, Mcal/kg	3.17	3.17	3.17	3.17	3.21	3.26	3.26	3.26	3.43	3.56	3.56	3.48
ME, Mcal/kg	2.60	2.60	2.60	2.60	2.63	2.67	2.67	2.67	2.81	2.92	2.92	2.85
NE_m_, Mcal/kg	1.69	1.69	1.69	1.69	1.72	1.75	1.75	1.75	1.87	1.97	1.97	1.91
NE_g_, Mcal/kg	1.08	1.09	1.09	1.08	1.12	1.15	1.15	1.13	1.24	1.33	1.33	1.27
Fatty acid, % total FA												
C16:0	20.21	20.95	21.07	20.58	19.50	20.66	20.92	20.25	17.08	17.00	17.44	17.43
C16:1 *trans-9*	0.51	0.52	0.51	0.51	0.49	0.50	0.49	0.49	0.37	0.34	0.32	0.36
C18:0	9.93	10.34	10.32	10.11	9.13	9.85	9.81	9.49	6.63	7.14	7.08	6.90
C18:1 *cis-9*	32.87	32.69	32.76	32.83	32.91	32.58	32.73	32.82	31.31	31.31	31.55	31.40
C18:2 *cis-9, cis*-12	25.56	24.38	24.23	24.98	27.26	25.40	25.08	26.09	35.15	34.90	34.36	34.51
C18:3 *cis-9, cis*-12, *cis*-15	1.38	1.40	1.38	1.37	1.52	1.49	1.46	1.50	1.66	1.23	1.17	1.51
SFA	35.24	36.60	36.71	35.89	33.33	35.58	35.82	34.64	27.02	27.79	28.19	27.86
MUFA	34.01	33.89	33.95	33.99	33.98	33.78	33.90	33.94	32.09	32.28	32.48	32.27
PUFA	26.94	25.78	25.61	26.34	28.78	26.89	26.53	27.58	36.80	36.12	35.53	36.02

ADP = adaptation diet offered from days 0 to 10 of the experiment; GRO = growing diet offered from days 11 to 35 of the experiment; FIN = finishing diet offered from days 36 to 108 of the experiment.

^*a*^DM = dry matter; NDF = neutral detergent fiber; peNDF = physically effective NDF; CP = crude protein; EE = ether extract; Ca = calcium; P = phosphorus; TDN = total digestible nutrients; DE = digestible energy; ME = metabolizable energy; NE_m_ = net energy for maintenance; NE_g_ = net energy for gain.

Given that, the overall objective of this experiment was to evaluate the effect of the source and FA profile of the supplements. All diets were formulated to be isolipidic, isonitrogenous, and isocaloric (Table 3). The experimental period lasted 108 d, whereas the adaptation, growing, and finishing diets were offered from days 0 to 10, 11 to 35, and 36 to 108, respectively. All diets were formulated using the Large Ruminant Nutrition System (LRNS; Fox et al., 2004) and formulated to result in an average daily gain (ADG) of approximately 1.5 kg/d. Diets were offered twice a day (0800 and 1600 h), in equal amounts, and all animals had ad libitum access to the diets and water. Throughout the experimental period, all animals were maintained in a roofed barn with concrete-based floors (7.5 m^2^/pen and 1.25 m of linear feedbunk/animal).

Seven days prior to beginning of the experiment, all animals were dewormed (Dectomax; Zoetis Saude Animal, São Paulo, SP, Brazil) and vaccinated against clostridium (Covexin-10; MSD Saude Animal, São Paulo, SP, Brazil). Moreover, from days −7 to −1 of the experimental design, all animals received 6 kg/animal per day of a diet containing (DM basis) 40% sugarcane bagasse, 20% ground corn, 20% citrus pulp, 17% peanut meal, and 3% mineral-vitamin mix. This management was performed to provide similar substrates and potentially equalize the rumen microbiome profile of the animals prior to treatment administration.

### Body Weight, Feed, and Blood Sampling

Individual animal shrunk BW was collected at the beginning (day 0) and end of the experimental period (day 108) after 16 h of feed and water withdrawal, in order to calculate BW change (final minus initial BW) and overall ADG during the experiment. Additional full BW measurements were taken every 28 ± 1 d (days 28, 57, and 85), in order to follow-up herd performance as the experiment progressed. Intermediate full BW, instead of shrunk BW, was obtained to prevent extreme dry matter intake (DMI) fluctuations following management and realimentation that could result in any potential rumen disorders (Allen et al., 2009) and to avoid the trigger of an acute-phase response that feed withdrawal might cause on the animals and subsequently impair feedlot performance and health (Marques et al., 2012, 2019).

Throughout the experimental period, total DMI was evaluated daily from each pen by collecting and weighing refusals. Samples of the offered and nonconsumed feed were collected daily from each pen and dried for 96 h at 50 °C in forced-air ovens for dry matter (DM) determination. Total DMI of each pen was divided by the number of animals within each pen and expressed as kilograms per animal per day. In addition, total DMI was expressed in % of BW by dividing the average DMI and average BW (initial + final) during the experiment. Calculation of ration total digestible nutrients (TDN) concentration was performed according to equations proposed by Weiss et al. (1992), and samples were analyzed in duplicates by wet chemistry procedures for concentrations of crude protein (CP), EE, and ash (AOAC, 1990). Neutral detergent fiber (NDF) was evaluated according to procedures described by Van Soest et al. (1991). Moreover, digestible energy (DE), metabolizable energy (ME), as well as calculations of net energy for maintenance (**NE**_**m**_) and gain (**NE**_**g**_) were performed according to equations described by NASEM (2016). Additionally, all feedstuffs used in the present experiment (Table 2) were analyzed for FA profile following procedures described in AOCS (2009) and according to total DMI, individual and total FA intake was calculated for each treatment and subsequently reported herein.

Dry matter intake fluctuation was performed according to the methodology described by Bevans et al. (2005), in which offered and nonconsumed feed were collected daily and the fluctuation was calculated as the DMI difference among 2 consecutive days. Moreover, at the end of the experiment, gain:feed (FE) ratio was calculated based on total BW gain and total DMI of the animals.

On days 0 and 108 of the experiment, blood samples were collected for plasma concentrations of blood lipoproteins. All blood samples were collected via jugular venipuncture into commercial blood collection tubes (Vacutainer, 10 mL; Becton Dickinson, Franklin Lakes, NJ). After collection, blood samples were centrifuged at 3,000 rpm for 15 min and stored at −20 °C in duplicates for subsequent laboratorial analyses. Commercial enzymatic kits (Bioclin; Belo Horizonte, MG, Brazil) were used for blood lipoprotein determination. The analyses were performed through Cobas Mira Plus device (Roche; São Paulo, SP, Brazil) and in addition to values ​​for total cholesterol, high-density lipoprotein (HDL), and triacylglycerols (TG), concentrations of very low-density lipoproteins (VLDL), and low-density lipoproteins (LDL) were also determined using the following equations (Friedewald et al., 1972):

*Equation 1.* VLDL = TG/5

*Equation 2.* LDL = total cholesterol – (HDL + VLDL)

### Ultrasound Evaluation

At the beginning and end of the experimental period (days 0 and 108, respectively), all animals enrolled into the experiment were submitted to ultrasound evaluations (Aloka SSD-500V with a 17.2 cm/3.50 MHz convex probe; Hitachi Healthcare Americas, Twinsburg, OH) and evaluations were performed by the same trained technician (DGT Brasil, Presidente Prudente, SP, Brazil). Evaluations were conducted according to procedures described by the Ultrasound Guidelines Council (UGC, 2014) and measurements of the ribeye area (REA), marbling, and backfat thickness (BFT) were collected on the *Longissimus thoracis* muscle between the 12th and 13th ribs and from the *Biceps femoris* muscle. Additionally, overall REA, marbling, and BFT gains were calculated as delta (Δ; final measurement − initial measurement) and the Δ was divided by the number of days in the experiment to represent the daily gain of these parameters (cm^2^ for REA, % for marbling, and mm for BFT of both tissues).

### Carcass Measurements

At the end of the experimental period, all animals were slaughtered following a waiting period of approximately 16 h, in a federally inspected commercial packing plant (JBS, Lins, SP, Brazil). Hot carcasses were separated into two symmetrical sections, weighed to obtain hot carcass weight (HCW), and individually identified. Dressing percent (DP) was calculated by dividing the HCW by final BW of the animal on day 108 of the experiment. Per standard industry procedures, initial DP of the animals was estimated in 50% and then it was calculated the amount of carcass gained by the animals during the experimental period (days 0 to 108). Carcass ADG was calculated by dividing the carcass gain and the number of days on feed (108 d), whereas yield gain (YG) was calculated by dividing carcass ADG (in kg) and ADG (in kg). Additionally, biological conversion (BC) was determined by dividing the total DMI of each pen by 15 kg of carcass produced during the entire experimental period.

### Meat Traits Evaluations

Following slaughter, all carcasses were cooled in a cold chamber for 48 h, whereas samples of *L. thoracis* muscle from the left carcasses were collected. For the colorimetric, weight losses due to cooking, and shear force analysis, three pieces of 2.54 cm each were collected proximally to the 12th and 13th ribs. All pieces were individually identified, vaccum-packed, and stored (−20 °C) until further laboratorial analysis.

#### Proximate composition

Moisture, CP, EE, and ashes were evaluated in samples collected from *L. thoracis* muscles. Moisture and CP contents were determined according to the methodologies previously described by AOAC (2007; methods 950.46 and 981.10, respectively). Ether extract and ash contents were also determined according to methods 920.39 and 920.153 reported on AOAC (2007).

#### Colorimetric, pH, weight loss due to cooking, and shear force

Upon analysis, samples were thawed in a refrigerator for 24 h and exposed to oxygen (O_2_) for 20 min. Following this procedure, meat pH was determined using a digital device (model HI-99163; Hanna Instruments Brasil, Barueri, SP, Brazil). For colorimetric parameters, the determination of the components L*, a*, and b* was performed according to Abularach et al. (1998). Color was evaluated on the surface of the samples using the CIE L*, a*, and b* system with a D65 illuminating and 10° as the standard evaluation point. A Minolta CM-2500-D device (Konica Minolta, Osaka, Japan) was used for color determination and calibrated with a blank standard sample. The following color parameters were used as follows: L* is a lightness index (0 = black and 100 = white), a* is the intensity of red color, an index that ranges from green (−) to red (+), and b* is the intensity of yellow color, an index ranging from blue (−) to yellow (+; Houben et al., 2000).

Following color measurement, *L. thoracis* samples were identified, weighed in a high-precision scale, and cooked at 170 °C until internal temperature reached 71 °C, as described by the American Meat Science Association (AMSA, 1995). Then, samples were cooled at room temperature and weighed in order to determine the weight loss due to cooking (final − initial weight; Honikel, 1998). The same samples were subsequently enveloped in a plastic film and placed on the refrigerator (4–6 °C) for 24 h. After 24 h, 6 to 8 cylinders of meat were collected from each sample (1.27 cm diameter each), parallel to the fibers. For shear force determination, the cylinders were sheared in a Warner–Bratzler Shear Force (WBSF) device with a 20 cm/min velocity, whereas the shear force of each sample was determined as the average of the 6 to 8 replicates and expressed as kg (Wheeler et al., 1997).

#### Meat FA profile

A subsample (approximately 2.8 g) of the *L. thoracis* muscle was used by adding it to a 50-mL Falcon tube (CRAL, Cotia, SP, Brazil). The extraction procedure followed the methodology described by Folch et al. (1957). Briefly, lipids were extracted by homogeneizing the sample with a 2:1 chloroform:metanol solution (Ultra Turrax; Marconi Equipamentos, Piracicaba, SP, Brazil) and isolated after the addition of a 1.5% NaCl solution. The isolated lipid was methylated and methyl esters were assembled according to the methodology described by Kramer et al. (1997). Fatty acids were quantified by gas chromatography (GC-2010 Plus Capillary GC; Shimadzu Scientific Instruments Inc., Columbia, MD) using a SP-2560 capilar column (100 m × 0.25 mm of diameter and 0.02 mm thickness; Supelco Inc., Bellefonte, PA). Initial column temperature was 45 °C with a progressive heating until temperature reached 175 °C, which was kept for 27 min. Then, temperature was increased at a rate of 4 °C/min until 215 °C, which was kept for 35 min. Hydrogen gas was used as the carrier with a 40 cm^3^/s flux. Fatty acids were identified using previously known and validated standards (Nu-Check Prep. Inc., Elysian, MN), quantified by evaluating the retetion time of the samples and normalization of the peak area of methyl esters (GS Solution 2.42 Software), whereas all FA were expressed as % of total known methyl esters.

### Tissue Gene Expression

#### RNA extraction, quantification, and integrity

Samples (*L. thoracis*) used for gene expression were randomly obtained from 1 animal/pen (6 animals/treatment) following slaughter at the packing plant, placed on tank containing liquid N, and stored in a biofreezer until further laboratorial analysis. Total RNA was extracted by using TRIzol (Thermo Fisher Scientific Inc., Waltham, MA) and manufacturer recommendations. Meat samples were frozen and milled (IKA T-25 Digital Ultra Tarrax, IKA Industrie, Königswinter, Germany) in 1 mL of TRIzol/100 mg of tissue. The homogenate was then transferred to a 1.5-mL tube and incubated during 5 min at room temperature. Choloroform was added (0.2 mL), tubes were vigorously shaken, incubated for 5 min at room temperature, and centrifuged at 12,000 *g* for 15 min at 4 °C. The aquous phase was discarded and the RNA was precipitated through the incubation of 0.5 mL of isopropyl alcohol for 10 min at room temperature. Following this step, the material was centrifuged at 12,000 *g* for 15 min at 4 °C and the RNA pellet was washed with 1 mL of 75% ethanol. The resultant material was again centrifuged at 12,000 *g* for 15 min at 4 °C and the supernatant was carefully removed, dried, and dissolved in a DNAse- and RNAse-free distilled water (Thermo Fisher Scientific, Inc.). Quantity and quality of isolated RNA were assessed via UV absorbance (NanoVue Plus, GE Healthcare, North Richland Hills, TX) at 260 mm and 260:280 nm ratio, respectively (Fleige and Pfaffl, 2006) and then stored at −80 °C until further processing. Furthermore, RNA integrity was evaluated through gel electrophoresis in 1% agarose gel, which was dissolved in a TBE-1X buffer (89 mM Tris, 89 mM boric acid, and 2 nM EDTA). An 1-µL RNA alíquota was added to a 5-µL buffer solution containing Orange-G and GelRed dye (Biotium, Fremont, CA). This mixture was applied into the gel and assigned to an 120V electrophoretic run for 90 min. The gel was recorded under an UV light and total RNA integrity was confirmed through the presence of ribosomal 28S and 18S reference bands.

#### DNAse treatment and reverse transcription

Total RNA was submitted to a DNAse treatment (DNAse-I Amplification Grade, Thermo Fischer Scientific, Inc.) in order to remove any DNA residue from the samples, following manufacturer recommendations. Reverse transcription of RNA was performed through the utilization of a commercial kit (High-Capacity cDNA archive kit, Thermo Fischer Scientific, Inc.), in which 1 µg of total RNA treated with DNAse was added to 2 µL of 10X RT buffer, 0.8 µL of 25X dNTP Mix, 1 µL of MultiScribe Reverse Transcriptase, 2 µL of 10X RT Random Primers, and 1 µL of RNase Inhibitor. The final volume of this reaction was adjusted to 20 µL with the UltraPure Distilled Watrer DNAse, RNAse Free (Thermo Fischer Scientific, Inc.). Each sample was incubated at 25 °C for 10 min, 37 °C for 120 min, and 85 °C for 5 min, whereas the final products of this reaction were stored at −20 °C and used in the polymerase chain reaction (PCR) analysis.

#### Real-time PCR

 Primer sets needed for gene amplification are described in Table 4 and were synthesized by Invitrogen (Carlsbad, CA), whereas cDNA samples were amplified using the GoTaq qPCR Master Mix (Promega, Madison, WI). For the analysis, 8 µL of diluted cDNA (1:20), 1.5 µL Forward Primer, 1.5 µL Reverse Primer, and 4 µL of the GoTaq were mixed. The reactions were performed in duplicates, as described: 95 °C for 10 min, followed by 40 cycles of denaturation at 95 °C for 15 s and annealing/extension at 60 °C for 1 min. The dissociation curve of the fragments was performed at the end of each PCR reaction in one 20-min step in which the temperature of the reaction gradually increases from 60 to 95 °C, allowing the specific evaluation of each primer by the presence of a unique fluorescence peak. Results were normalized to the genes of interest (*β-actin* and *glyceraldehyde-3-phosphate dehydrogenase*) and expressed as relative fold change (2^−ΔΔCT^), as described by Livak and Schmittgen (2001). Genes of interest and analyzed herein include peroxisome proliferator-activated receptor-γ (PPAR-γ), sterol regulatory element-binding protein-1 (SREBP-1), stearoyl-CoA desaturase-1 (SCD), and lipoprotein lipase (LPL; Table 4).

**Table 4.** Target gene, primer sequence (forward and reverse), and accession number for all gene transcripts analyzed by real-time PCR

Target gene	Primer sequence	Accession no.
Peroxisome proliferator-activated receptor-γ		NM_181024.2
* Forward*	GTGAAGTTCAACGCACTGGA	
* Reverse*	ATGTCCTCAATGGGCTTCAC	
Sterol regulatory element-binding protein-1		NM_001113302.1
* Forward*	ACCGCTCTTCCATCAATGAC	
* Reverse*	GCTGAAGGAAGCGGATGTAG	
Stearoyl-CoA dessaturase-1		NM_173959.4
* Forward*	GCCAGAGTTGGGTCAAACAT	
* Reverse*	CTCTTCTTCAAGCCCCCTCT	
Lipoprotein lipase		NM_001075120.1
* Forward*	CTCCTGATGATGCGGATTTT	
* Reverse*	ACATCCTGGTTGGAAAGTGC	
Beta-actin		BC142413.1
* Forward*	GATGAGATTGGCATGGCTTT	
* Reverse*	GTCACCTTCACCGTTCCAGT	
Glyceraldehyde-3-phosphate dehydrogenase		NM_001034034.1
* Forward*	TGACCCCTTCATTGACCTTC	
* Reverse*	GATCTCGCTCCTGGAAGATG	

### Meat Sensorial Analysis

In the present experiment, *L. thoracis* steaks were used for sensorial analysis, which was conducted by the University of São Paulo with 115 nontrained customers from a community located in Pirassununga, SP, Brazil. *Longissimus thoracis* steaks were thawed at room temperature, placed in an aluminum tray, taken to an industrial oven (170 °C), heated until internal temperature reached 40 °C, turned in the tray inside the oven, and kept in the oven until internal temperature of the steak reached 71 °C (AMSA, 1995). After oven removal, steaks were rectangularly sliced (2.5 × 1.5 × 1.5 cm), individually packed, and maintained in a stove at 60 °C until the beginning of the sensorial analysis tests. One sample representing one steak from each treatment was offered at each time to customers. The sequence by which samples were offered to customers was balanced, in order to minimize the effect that sequence could have on customers judgement and proper results assessment. Samples were packed into an aluminum foil and offered inside previously identified plastic cups (A–D).

For sensorial analysis data, softness, juiciness, regular flavor and odors, as well as atypical flavor and aroma were evaluated according to methodologies previously reported by AMSA (1995). For softness, juiciness, and regular flavor and odor evaluations, customers filled out a form with the answers in a 9-point scale, where 1 = extremely dissatisfied and 9 = extremely satisfied, exception. For the atypical meat features (atypical flavor and aroma), a 5-point scale was used, where 1 = extremely present and 5 = absent (Meilgaard et al., 1999).

### Statistical Analysis

For all analyses performed herein, pen was considered the experimental unit. All data were analyzed using the MIXED procedure of SAS (Version 9.4; SAS Inst., Inc.; Cary, NC) and the Satterthwaite approximation to determine the denominator df for the test of fixed effects. The model statement used for all analyses contained the fixed effects of treatment. Data were analyzed using block, animal (pen), and pen (treatment) as random variables. Blood samples and US measurements obtained on day 0 were analyzed for covariates and final results obtained on day 108 were covariately adjusted. All results are reported as least square means and separated using the PDIFF. Significance was set at *P* ≤ 0.05 and tendencies were denoted if *P* > 0.05 and *P* ≤ 0.10. Throughout the paper, results are reported according to the main effects.

## RESULTS

### Intake, Performance, and Blood Profile

As expected, no treatment effects were observed on initial BW (*P* = 0.99), indicating that animals were managed similarly prior to the beginning of the experiment (Table 5). At the end of the experimental period, BW, ADG, DMI, DMI as % of BW, DMI fluctuation, and FE did not differ among treatments (*P* ≥ 0.30; Table 5). Moreover, analysis of BW and ADG data obtained from intermediate time points on days 28, 57, and 85 of the experimental period did not differ among treatments (*P* ≥ 0.30; data not shown).

**Table 5.** Performance of *Bos indicus* beef animals offered diets containing whole cottonseed and corn germ (CONT; *n* = 6), calcium salts of soybean oil (CSSO; *n* = 6), calcium salts of palm, soybean, and cottonseed oil (CSVO; *n* = 6), or a mixture of whole cottonseed, corn germ, and CSVO (MIXT; *n* = 6) as lipid sources

Item	Treatments				SEM	*P*
	CONT	CSSO	CSVO	MIXT		
Initial BW, kg	399.9	400.3	400.0	399.7	0.33	0.60
Final BW, kg	555.5	550.4	545.5	545.3	6.08	0.61
ADG, kg^*a*^	1.44	1.39	1.35	1.35	0.056	0.62
DMI						
kg/d	10.10	10.03	10.12	10.00	0.224	0.98
% BW	2.10	2.12	2.15	2.12	0.037	0.81
Fluctuation, kg/d^*b*^	5.69	5.32	5.73	6.05	0.349	0.61
FE, g/kg	141	142	141	142	3.2	0.98

Experimental diets were offered for 108 d.

^*a*^Calculated by the difference of initial and final BW collected on days 0 and 108, respectively, after 16 h of feed and water withdrawal.

^*b*^Calculated according to methodology described by Bevans et al. (2005).

Neutral detergent fiber, physically effective NDF (peNDF), and starch intakes were affected by treatments offered herein (*P* < 0.01), given that CONT and MIXT had a greater intake of NDF and peNDF when compared with cohorts offered CSSO and CSVO (*P* < 0.03; Table 6). On the other hand, CSSO and CSVO supplementation yielded greater starch intake vs. CONT and MIXT (*P* < 0.01), but MIXT also resulted in greater starch intake vs. CONT (*P* < 0.001; Table 6). No further treatment effects were observed on CP, EE, TDN, and NE_g_ intakes (*P* ≥ 0.36; Table 6). For the individual FA intake evaluation, treatment differences were observed on C10:0, C16:1 *trans*-9, C18:1 *cis*-9, C18:2, C18:3, C22:0, C24:0, total PUFA, and ratios (*P* ≤ 0.05; Table 6), whereas tendencies were observed on C12:0, C16:0, C18:0, C20:0, total FA, total SFA, and total MUFA (*P* ≤ 0.10; Table 6). More specifically, animals receiving CONT had a greater C10:0, C16:0, C16:1 *trans*-9, C18:1 *cis*-9, C18:2, C18:3, total FA, MUFA, and PUFA vs. CSSO and MIXT (*P* < 0.05), whereas CSVO had interemediate values for some of these FA (Table 6). Conversely, intake ratios of SFA:MUFA and SFA:PUFA were all reduced for CONT vs. other treatments (Table 6).

**Table 6.** Nutrient intake of *Bos indicus* beef animals offered diets containing whole cottonseed and corn germ (CONT; *n* = 6), calcium salts of soybean oil (CSSO; *n* = 6), calcium salts of palm, soybean, and cottonseed oil (CSVO; *n* = 6), or a mixture of whole cottonseed, corn germ, and CSVO (MIXT; *n* = 6) as lipid sources

Item^*a*^	Treatments				SEM	*P*
	CONT	CSSO	CSVO	MIXT		
DMI, kg/d	10.02	9.84	10.06	9.92	0.254	0.93
DMI, % BW	2.10	2.09	2.13	2.12	0.033	0.83
Nutrient intake, kg/d						
CP	1.44	1.42	1.45	1.43	0.036	0.95
NDF	2.89^a^	2.54^b^	2.60^b^	2.77^a^	0.068	< 0.01
peNDF	1.76^a^	1.33^c^	1.36^c^	1.66^b^	0.038	< 0.0001
EE	0.50	0.49	0.50	0.49	0.013	0.88
TDN	7.65	7.74	7.89	7.64	0.196	0.78
Starch	2.77^c^	3.66^a^	3.73^a^	3.28^b^	0.090	< 0.0001
NEg, Mcal/kg	12.02	12.47	12.72	12.06	0.313	0.36
Fatty acid, g/d						
C10:0	0.77^a^	0.66^b^	0.70^b^	0.66^b^	0.019	0.01
C12:0	5.84^bc^	6.11^b^	6.44^ab^	6.18^b^	0.135	0.10
C14:0	6.71	6.48	6.64	6.20	0.141	0.19
C16:0	190.88^a^	179.79^bc^	186.10^ab^	172.03^c^	4.161	0.09
C16:1 *trans-9*	4.37^a^	3.86^b^	3.79^b^	3.77^b^	0.085	< 0.01
C17:0	1.62	1.60	1.58	1.47	0.037	0.12
C18:0	109.93^ab^	114.46^a^	114.26^a^	102.56^b^	2.631	0.06
C18:1 *cis-9*	336.99^a^	313.02^bc^	319.25^ab^	298.16^c^	7.387	0.05
C18:2 *cis-9, cis*-12	344.48^a^	311.91^b^	311.37^b^	295.91^b^	7.913	0.02
C18:3 *cis-9, cis*-12, *cis*-15	17.24^a^	12.88^c^	12.57^c^	13.96^b^	0.334	< 0.0001
C20:0	9.08^a^	8.66^ab^	8.73^ab^	8.13^b^	0.191	0.08
C20:1	4.90	4.91	4.95	4.53	0.104	0.11
C22:0	9.07^b^	10.07^a^	10.18^a^	8.77^b^	0.204	< 0.01
C24:0	6.72^b^	7.27^a^	7.36^a^	6.38^b^	0.154	< 0.01
Total	1,052.24^a^	984.38^bc^	996.31^ab^	930.61^c^	23.541	0.06
SFA	340.99^a^	335.14^ab^	341.98^a^	312.07^b^	7.689	0.10
MUFA	346.26^a^	323.39^b^	329.34^ab^	307.09^b^	7.601	0.06
PUFA	361.72^a^	324.80^b^	323.95^b^	309.86^b^	8.239	0.01
SFA:MUFA ratio	0.984^c^	1.034^a^	1.036^a^	1.015^b^	0.0008	< 0.0001
SFA:PUFA ratio	0.967^d^	1.056^b^	1.081^a^	1.030^c^	0.0015	< 0.0001

Experimental diets were offered for 108 d. Within rows, values with different superscript differ (*P* ≤ 0.10).

^*a*^Dry matter and nutrient intakes were evaluated on a daily basis throughout the experimental period (days 0–108) and calculated based on amount of ration offered and refusal on the bunk.

For blood variables, samples collected on day 0 differed among treatments (*P* < 0.01) for TG and VLDL and tended to differ for LDL (*P* = 0.07; Table 7). Plasma TG and VLDL were reduced for CONT vs. other treatments (*P* ≤ 0.02), whereas LDL concentrations was greater for CSSO vs. CONT and CSVO (*P* ≤ 0.04), with MIXT being intermediate (*P* ≥ 0.17; Table 7). Although differences were observed on day 0 of the experiment, only tendencies were observed for a covariate effect on TG (*P* = 0.09) and VLDL (*P* = 0.07). At the end of the experimental period, blood concentrations of TG and VLDL were reduced for CONT-supplemented animals vs. all other treatments (*P* < 0.05; Table 7). Additionally, TG concentrations were also greater for CSSO vs. MIXT (*P* = 0.04), whereas no other differences were detected for TG and VLDL (*P* ≥ 0.16; Table 7).

**Table 7.** Blood profile of *Bos indicus* beef animals offered diets containing whole cottonseed and corn germ (CONT; *n* = 6), calcium salts of soybean oil (CSSO; *n* = 6), calcium salts of palm, soybean, and cottonseed oil (CSVO; *n* = 6), or a mixture of whole cottonseed, corn germ, and CSVO (MIXT; *n* = 6) as lipid sources

Item^*a*^	Treatments				SEM	*P*
	CONT	CSSO	CSVO	MIXT		
Cholesterol, mg/dL						
Initial	171.0	183.4	177.7	187.9	5.52	0.20
Final	253.7	258.2	254.3	265.6	13.64	0.94
TG, mg/dL						
Initial	8.8^a^	12.5^b^	11.8^b^	11.4^b^	0.69	< 0.01
Final	15.0^c^	25.4^a^	22.1^ab^	20.4^b^	1.70	0.03
HDL, mg/dL						
Initial	84.9	85.9	88.0	89.3	2.00	0.44
Final	105.7	104.6	106.0	103.1	2.88	0.89
VLDL, mg/dL						
Initial	1.8^a^	2.5^b^	2.4^b^	2.3^b^	0.14	< 0.01
Final	3.0^b^	4.9^a^	4.4^a^	4.1^a^	0.34	0.04
LDL, mg/dL						
Initial	83.1^b^	107.5^a^	86.8^b^	94.4^ab^	6.39	0.07
Final	144.3	138.0	145.3	162.5	13.45	0.58

Experimental diets were offered for 108 d. Blood samples were collected on days 0 and 108 of the experimental period. Values obtained on day 0 were analyzed as covariates and final results (day 108) were covaritely adjusted. Within rows, values with different superscript differ (*P* ≤ 0.10).

^*a*^TG = triglycerides; HDL = high-density lipoprotein; VLDL = very low-density lipoprotein; LDL = low-density lipoprotein.

### Ultrasound Measurements and Carcass Characteristics

For ultrasound measurements, values obtained on day 0 were significant covariates for REA (*P* = 0.03) and BFT of *B. femoris* (*P* = 0.01), but did not differ among treatments (*P* ≥ 0.20; Table 8). At the end of the experimental period, no treatment differences were observed for any parameter of carcass obtained through US, such as final measurements of REA, BFT for *L. thoracis* and *B. femoris*, and marbling, Δ, and daily gains (*P* ≥ 0.27; Table 8). Furthermore, following slaughter, carcasses were evaluated and no treatment effects were observed for any of the parameters evaluated herein (*P* ≥ 0.22; Table 8).

**Table 8.** Ultrasound measurements (*L. thoracis* and *B. femoris*) and carcass characteristics of *Bos indicus* beef animals offered diets containing whole cottonseed and corn germ (CONT; *n* = 6), calcium salts of soybean oil (CSSO; *n* = 6), calcium salts of palm, soybean, and cottonseed oil (CSVO; *n* = 6), or a mixture of whole cottonseed, corn germ, and CSVO (MIXT; *n* = 6) as lipid sources

Item	Treatments				SEM	*P*
	CONT	CSSO	CSVO	MIXT		
Ultrasound measurements						
REA, cm^2^						
Initial	70.7	68.6	67.7	68.1	1.39	0.43
Final	82.7	81.1	80.9	80.6	1.54	0.79
Δ	13.2	12.2	12.3	11.9	1.53	0.94
Daily gain	0.122	0.113	0.114	0.111	0.0138	0.95
L. thoracis BFT, mm						
Initial	2.29	2.44	2.37	2.38	0.053	0.32
Final	5.32	5.30	5.27	5.99	0.339	0.37
Δ	2.87	2.99	2.90	3.62	0.313	0.32
Daily gain	0.027	0.028	0.027	0.033	0.0029	0.34
Marbling, %						
Initial	2.01	2.05	2.04	2.12	0.125	0.94
Final	2.93	2.71	3.00	2.92	0.100	0.61
Δ	0.82	0.70	0.88	0.77	0.114	0.73
*B. femoris* BFT, mm						
Initial	2.77	3.18	3.04	3.11	0.136	0.20
Final	7.46	7.79	7.60	8.11	0.344	0.59
Δ	4.23	4.92	4.57	5.13	0.325	0.27
Daily gain	0.039	0.046	0.042	0.047	0.0030	0.27
Carcass traits^*a*^						
DP, %	55.95	55.23	55.93	55.78	0.35	0.52
HCW, kg	310.8	303.9	303.9	304.2	3.73	0.51
Carcass ADG, kg	1.026	0.960	0.962	0.966	0.0351	0.51
YG, %	71.3	69.0	71.6	71.8	1.44	0.59
BC, DMI/15-kg carcass	147.7	156.4	158.3	155.4	3.59	0.22

Experimental diets were offered for 108 d. Within rows, values with different superscript differ (*P* ≤ 0.10). Ultrasound evaluations were performed on days 0 (initial) and 108 (final) of the experimental period.

^*a*^DP = dressing percent; HCW = hot carcass weight; YG = yield gain; BC = biological conversion.

### Meat Traits and FA Profile

No treatment effects were observed for any of the proximate analysis, meat pH, weight loss due to cooking, and WBSF (*P* ≥ 0.13; Table 9). On the other hand, treatments offered during the experimental period altered colorimetric parameters in a manner that meat obtained from CONT animals had greater L*, a*, and b* values vs. cohorts offered MIXT (*P* < 0.01), whereas CSSO and CSVO groups had interemediate values for L* and a*, which did not differ from the other treatments (*P* ≥ 0.16; Table 9).

**Table 9.** Meat characteristics of *Bos indicus* beef animals offered diets containing whole cottonseed and corn germ (CONT; *n* = 6), calcium salts of soybean oil (CSSO; *n* = 6), calcium salts of palm, soybean, and cottonseed oil (CSVO; *n* = 6), or a mixture of whole cottonseed, corn germ, and CSVO (MIXT; *n* = 6) as lipid sources

Item	Treatments				SEM	*P*
	CONT	CSSO	CSVO	MIXT		
pH	5.52	5.63	5.51	5.57	0.05	0.46
Proximate analysis, %						
Moisture	74.4	74.7	74.8	75.1	0.28	0.31
CP	22.7	22.6	22.8	22.6	0.32	0.75
EE	1.3	1.3	1.8	1.2	0.18	0.13
Ash	1.1	1.1	1.1	1.1	0.01	0.32
Colorimetric parameters^*a*^						
L*	37.7^a^	35.8^ab^	35.9^ab^	35.1^b^	0.63	0.04
a*	18.0^a^	16.8^ab^	17.4^ab^	16.2^b^	0.39	< 0.01
b*	14.7^a^	13.1^b^	13.5^ab^	12.7^b^	0;44	0.01
Cooking loss, %	29.2	28.5	28.7	30.2	1.02	0.66
WBSF, kg^*b*^	3.7	4.0	3.6	4.2	0.24	0.38

Experimental diets were offered for 108 d. Within rows, values with different superscript differ (*P* ≤ 0.10).

^*a*^L* = lightness index (0 = black and 100 = white); a* = intensity of red color, an index that ranges from green (−) to red (+); b* = intensity of yellow color, an index ranging from blue (−) to yellow (+; Houben et al., 2000)

^*b*^WBSF = Warner–Bratzler Shear Force.

A complete description of the meat FA profile obtained in the present experiment is reported in Table 10. In agreement with differences aforementioned on FA intake, experimental treatments offered herein for finishing *Bos indicus* beef animals affected meat concentrations of C18:0, C18:1 *trans*-9, C18:1 *trans*-10, C18:1 *trans*-11, C18:2, C18:2 *cis*-7, *trans*-9 CLA, and C18:2 *cis*-9, *trans*-11, and total SFA (*P* < 0.02; Table 10). Supplementation with CONT increased C18:0 vs. supplementation with calcium salts as the unique lipid source of the diets (*P* < 0.02), whereas no differences were observed among CONT vs. MIXT and calcium salts of fatty acids vs. MIXT (*P* ≥ 0.11; Table 10). On the other hand, supplementation with CSSO yielded greater meat concentrations of C18:1 *trans*-10, C18:2 *cis*-7, *trans*-9 CLA, and C18:2 *cis*-9, *trans*-11 CLA vs. other treatments (*P* < 0.01; Table 10). Additionally, meat from animals supplemented with MIXT also had greater concentrations of C18:2 *cis*-7, *trans*-9 CLA vs. CONT and CSVO (*P* < 0.05; Table 10). No other treatment effects were observed for the other individual FA, total SFA, MUFA, PUFA, and the FA ratios (*P* ≥ 0.11; Table 10).

**Table 10.** Meat FA profile of *Bos indicus* beef animals offered diets containing whole cottonseed and corn germ (CONT; *n* = 6), calcium salts of soybean oil (CSSO; *n* = 6), calcium salts of palm, soybean, and cottonseed oil (CSVO; *n* = 6), or a mixture of whole cottonseed, corn germ, and CSVO (MIXT; *n* = 6) as lipid sources

Fatty acid, % total FA^*a*^	Treatments				SEM	*P*
	CONT	CSSO	CSVO	MIXT		
C10:0	0.062	0.083	0.043	0.066	0.0152	0.34
C12:0	0.061	0.064	0.055	0.069	0.0056	0.38
C14:0	2.647	2.564	2.599	2.791	0.1336	0.65
C14:1 *cis-9*	0.507	0.587	0.563	0.594	0.0350	0.31
C15:0	0.318	0.324	0.310	0.356	0.0162	0.24
C15:1 *cis*-10	0.096	0.108	0.137	0.080	0.0175	0.16
C16:0	23.249	23.178	23.845	24.011	0.5168	0.59
C16:1 *trans-9*	0.311	0.323	0.285	0.323	0.0118	0.11
C16:1 *cis*-10	2.286	2.232	2.478	2.484	0.1435	0.50
C17:0	0.810	0.836	0.801	0.848	0.0329	0.72
C17:1 *cis*-10	0.561	0.482	0.609	0.440	0.0812	0.47
C18:0	17.079^a^	14.516^b^	14.719^b^	16.166^ab^	0.5849	0.02
C18:1 *trans-9*	0.293	0.482	0.283	0.291	0.0692	0.16
C18:1 *cis-9*	34.629	34.305	33.455	35.056	1.0544	0.75
C18:1 *trans-10*	0.507^b^	1.097^a^	0.625^b^	0.588^b^	0.0986	< 0.01
C18:1 *trans-11*	1.423	1.501	1.192	1.455	0.1182	0.28
C18:2 *cis-9, cis*-12	4.437	5.278	6.291	4.430	0.7647	0.29
C18:2 *cis-7, trans-9 CLA*	0.052^c^	0.097^a^	0.059^c^	0.079^b^	0.0064	< 0.001
C18:2 *cis-9, trans-11 CLA*	0.326^b^	0.450^a^	0.299^b^	0.331^b^	0.0282	< 0.01
C18:3 *cis-9, cis*-12, *cis*-15	0.235	0.263	0.285	0.261	0.0275	0.66
C20:4	0.716	0.848	1.084	0.653	0.1878	0.40
C20:5	0.133	0.139	0.206	0.102	0.0365	0.26
C22:5	0.304	0.365	0.461	0.287	0.0738	0.36
SFA	44.245	41.565	42.371	44.306	1.0383	0.19
MUFA	40.612	41.117	39.629	41.312	1.1260	0.72
PUFA	6.201	7.439	8.684	6.144	1.0233	0.28
n-3	0.893	0.891	0.893	0.894	0.0082	0.99
n-6	8.545	8.585	8.552	8.570	0.6481	1.00
n-6:n3 ratio	7.426	6.949	5.352	7.293	0.8668	0.33
Unknown	8.942	9.879	9.316	8.238	0.9874	0.65
SFA:MUFA ratio	1.089	1.018	1.075	1.076	0.0391	0.59
SFA:PUFA ratio	5.248	5.099	5.302	4.577	0.4545	0.67

^*a*^Within rows, values with different superscript differ (*P* ≤ 0.10).

### Tissue Gene Expression

**Fig. 1.**
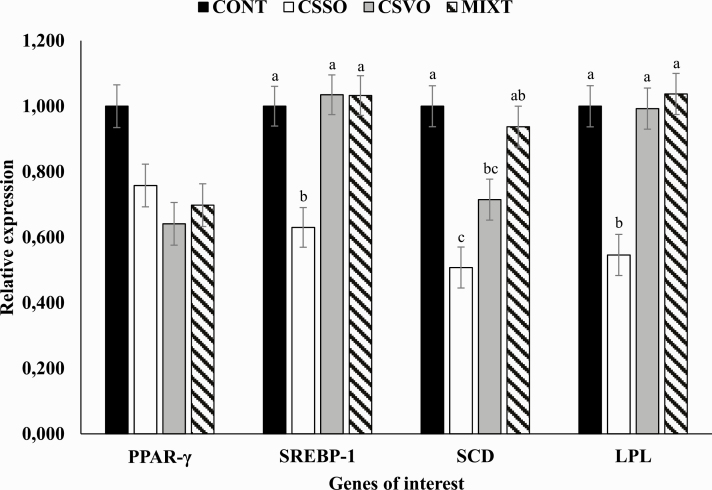
Relative expression meat genes associated with lipid metabolism of *Bos indicus* beef animals offered diets containing whole cottonseed and corn germ (CONT; *n* = 6), calcium salts of soybean oil (CSSO; *n* = 6), calcium salts of palm, soybean, and cottonseed oil (CSVO; *n* = 6), or a mixture of whole cottonseed, corn germ, and CSVO (MIXT; *n* = 6) as lipid sources. Within genes, values with different letters represent treatment differences (*P* ≤ 0.10).

From the genes analyzed herein (Table 4), treatment effects were detected on SREBP-1, SCD, and LPL (*P* < 0.03; Fig. 1), whereas no statistical differences were observed on PPAR-γ (*P* = 0.23; Fig. 1). For all genes that statistically differed among treatments, a downregulation was observed when animals were supplemented with CSSO (*P* < 0.05). Supplementation with CSVO also reduced SCD expression vs. CONT (*P* = 0.05), whereas no differences were observed between CSVO and MIXT (*P* = 0.12; Fig. 1). Nonetheless, no other treatment differences were observed on SREBP-1 and LPL gene expression among CONT, CSVO, and MIXT (*P* ≥ 0.81; Fig. 1).

### Meat Sensorial Analysis

Treatment effects were observed on regular flavor (*P* = 0.01) in a manner that supplementation with CSSO yielded greater results compared with CONT, CSVO, and MIXT (*P* ≤ 0.05; Table 11). Additionally, CSSO supplementation resulted in greater (*P* = 0.05) aroma results vs. CONT and CSVO, whereas MIXT values were intermediate compared with CSSO (*P* = 0.57), as well as with CONT and CSVO (*P* ≥ 0.11 Table 11). No other treatment effects were observed for softness and juiciness, as well as atypical flavor and aroma (*P* ≥ 0.17; Table 11).

**Table 11.** Sensorial analysis of meats obtained from *Bos indicus* beef animals offered diets containing whole cottonseed and corn germ (CONT; *n* = 6), calcium salts of soybean oil (CSSO; *n* = 6), calcium salts of palm, soybean, and cottonseed oil (CSVO; *n* = 6), or a mixture of whole cottonseed, corn germ, and CSVO (MIXT; *n* = 6) as lipid sources

Item	Treatments				SEM	*P*
	CONT	CSSO	CSVO	MIXT		
Softness	7.21	6.95	7.18	7.18	0.170	0.69
Juiciness	6.89	7.00	6.90	7.04	0.156	0.88
Regular flavor	7.00^bc^	7.48^a^	6.81^c^	7.18^b^	0.149	0.01
Regular aroma	6.66^b^	7.15^a^	6.69^b^	7.03^ab^	0.151	0.05
Atypical flavor	4.29	4.47	4.36	4.38	0.088	0.53
Atypical aroma	4.31	4.44	4.35	4.43	0.092	0.72

Experimental diets were offered for 108 d. Within rows, values with different superscript differ (*P* ≤ 0.10).

## DISCUSSION

The primary goal of the present experiment was to evaluate the effects of lipid feedstuff source and FA profile offered to *Bos indicus* finishing bulls consuming isonitrogenous, isocaloric, and isolipidic high-concentrate diets. Therefore, lipid sources used herein included whole cottonseed, corn germ, as well as calcium salts of fatty acids based on soybean oil or a mixture of palm, soybean, and cottonseed oils. Based on this rationale, not only the source, but also the FA profile of the supplements would be different, as reported in Table 1 (range of SFA, MUFA, and PUFA contents were 15.7–38.3%, 31.4–43.7%, and 25.3–45.2%, respectively). It was hypothesized that the different sources of lipids could differently affect the ruminal environment, given that the availability of the oils would differ and consequently, the biohydrogenation process would likely alter the FA profile reaching the small intestine for subsequent absorption and tissue incorporation. One might question the lack of a negative control group in the present experiment, but this strategy was adopted considering that the benefits regarding the utilization of lipids in feedlot diets have already been reported by several research groups (NASEM, 2016), whereas possible effects of the source and FA profile of lipid ingredients for beef cattle are scarce and still lacking in the literature.

No differences were observed among treatments on total DMI (as kg/d or % BW), whereas NDF and peNDF intakes were reduced due to CSSO and CSVO supplementation. These differences might be related to the greater content of dietary NDF and peNDF in CONT vs. CSSO and CSVO, whereas no differences were observed in nutrients offered in similar amounts (CP, TDN, and NE_g_) and demonstrating the effectiveness of the experimental design in providing isonitrogenous, isocaloric, and isolipic diets to the herd. Similarly, treatment differences reported on starch intake agree with nutrient composition of the diets, given that starch is the main component of corn grains (Huntington, 1997) and, consequently, diets containing greater amounts of corn result in greater starch intakes (Cappellozza et al., 2014). Evaluating lipid supplements with different sources and FA profile to cattle, Fiorentini et al. (2014) reported a reduction in DM, OM, CP, and NDF intakes when lipid sources were not ruminally protected vs. nonlipid or ruminally protected cohorts. On the other hand, inclusion of a supplement similar to the CSSO utilized herein did not affect intake of any of the nutrients aforementioned when compared with the negative control group (nonlipid-supplemented). In dairy cattle, de Souza et al. (2017) reported that supplementation with calcium salts of palm oil to grazing dairy cows improved milk production, fat-corrected milk, and milk production efficiency vs. cohorts supplemented with calcium salts of soybean oil, suggesting that FA profile of the supplement plays a major role on productive parameters of ruminants.

In agreement with our experimental design, treatment differences were observed on intakes of several FA, such as C16:0, C18:0, C18:1 *cis-9,* C18:2, and C18:3, as well as total FA, MUFA, PUFA, SFA:MUFA, and SFA:PUFA ratios. The effects of C18:0 and C18:1 *cis*-9 on metabolism and productive responses of dairy cattle receiving soybean hulls or whole cottonseed have been reported by de Souza et al. (2018). Overall, supplementation of a mixture of C16:0 and C18:0 FA improved DMI and milk fat and protein content vs. a mixture of C16:0 and C18:1 FA, whereas opposite results were observed on nutrient (DM, NDF, FA, C16, and C18 FA) digestibility and BW/BCS changes. Nonetheless, these effects of FA profile were more significant when diets contained soybean hulls vs. whole cottonseed, likely indicating that quality and type of fiber might affect these results. In de Souza et al. (2018), FA intakes ranged from 150 to 180 g/d for C16 and C18 FA, whereas in our experiment these values ranged from 12 to 38 g/d. Therefore, it might be speculated that differences on FA intakes reported herein were not large enough to provide similar treatment effects on DMI as reported by de Souza et al. (2018).

Greater hypophagic effects have been reported when unsaturated vs. saturated C18 FA were offered in the diets of lactating dairy cows (Harvartine and Allen, 2006), primarily due to the faster and greater oxidation rate in bovine hepatocytes (Mashek and Grummer, 2003). In the present experiment, CONT animals had greater C18:1 *cis*-9, C18:2, C18:3, and total PUFA intake, which did not result in a reduction in DMI. Nonetheless, ruminal biohydrogenation was not evaluated or estimated herein, and one might speculate that a greater biohydrogenation, C18:2 CLA formation and flow to the duodenum, as well as consequent FA profile alteration occurred in CONT animals, whereas calcium salts of fatty acids are manufactured and used in beef cattle diets as a manner to reduce the rate at which ruminal biohydrogenation occurs (Sukhija and Palmquist, 1990) and, therefore, reduce the potential negative effect of unsaturated FA in the rumen (Jenkins et al., 2008). On the other hand, in animals receiving calcium salts of fatty acids, a greater hypophagic effect might have occurred, as proposed by Harvartine and Allen (2006), given that it would be expected that a greater amount of PUFA would reach the small intestine. Hence, one might speculate that ruminal biohydrogenation and hypophagic effect counteracted and resulted in similar DMI. Based on this rationale, it is important to mention that one pitfall of the present experiment is the absence of the blood FA profile of the animals, given that this parameter usually reflects the duodenal flow of FA in ruminants (Archibeque et al., 2005; Hess et al., 2008). Nonetheless, in a manner to mitigate this factor, tissue expression of enzymes involved in lipid metabolism and carcass characteristics that have been shown to alter FA profile of blood and meat of ruminants (Scholljegerdes et al., 2007), as well as meat FA profile were evaluated.

The lack of differences on productive responses during the feedlot phase is in agreement with the fact that diets were formulated to be isocaloric, isonitrogenous, and isolipidic. Similarly, no treatment differences were observed on carcass characteristics and US measurements of animals slaughtered at the end of the experimental period. In agreement with our results, Fiorentini et al. (2014) also observed similar ADG, FE, and carcass characteristics of beef animals receiving a 60:40 roughage:concentrate diet containing calcium salts of soybean oil or whole soybeans. Moreover, Schubach et al. (2019) also reported similar performance and carcass characteristics when different sources of calcium salts of fatty acids (palm- and soybean oil-based) were strategically supplied to cattle on creep-feeding (60 d) and/or preconditioning (40 d). Conversely, Nascimento (2017) reported greater HCW, BFT, and REA of *B. indicus* beef animals offered CSVO vs. CSSO during feedlot for a period of 140 d. Cooke et al. (2011) also reported greater marbling in animals supplemented with a source of calcium salts of soybean oil for 28 d during preconditioning prior to transport to feedlot. Therefore, further research is warranted to determine the possible effects of specific FA, saturation degree, dose, and supplementation length on performance responses and carcass characteristics of *B. indicus* finishing beef cattle.

In cattle, Noro and Kobayashi (1995) hypothesized that cholesterol is related to the development of adipose tissue and its content in adipose tissue is positively correlated to the volume of adipose tissue. Furthermore, these authors demonstrated that the levels of marbling in beef cattle might be inversely correlated to HDL levels and directly related to LDL. The primary site of endogenous cholesterol synthesis is the small intestine and adipose tissue, with the liver producing a small proportion of the total endogenous cholesterol (Liepa et al., 1978). Lipid supplementation has been shown to stimulate synthesis and accumulation of cholesterol and lipoproteins in tissues and body fluids of ruminants (Williams and Stanko, 2000), whereas the effects of FA profile on concentrations of these compounds are scarce in the literature. Previous studies reported possible effects of FA on cholesterol and lipoprotein concentrations in beef cattle. As an example, supplementation with lipid sources rich in SFA usually results in an inhibition on mRNA expression of LDL receptors, consequently increasing plasma LDL concentrations (Mustad et al., 1997; Boekholdt et al., 2006). In the present experiment, inclusion of calcium salts of fatty acids in the diets of finishing beef animals resulted in greater blood concentrations of TG and VLDL, whereas no effects were observed on cholesterol, HDL, and LDL. Hocquette et al. (2010) reported that, in cattle, fat deposition is linked with genetic patterns of individuals, whereas marbling is a balance between absorption, synthesis, and degradation of TG. Although plasma TG concentrations were observed on calcium salts of fatty acids-supplemented animals, no beneficial effects were observed on marbling. It could be speculated that although more starch was consumed by animals receiving CSSO, CSVO, and MIXT diets, more PUFA also reached the small intestine for absorption and consequently reduced the activity of enzymes involved in adipocytes differentiation, but this is not in complete agreement with the data reported herein for marbling and mRNA expression of enzymes. Hence, the reason for the observed results on TG and VLDL, even in an experimental design of similar EE intakes, is not known and deserve further investigation.

Ether extract content is positively correlated with marbling (Moore et al., 2010) and meat from Nellore bulls would be classified as having a small amount of marbling based on EE values. In another recent trial, Teixeira et al. (2017) reported breed differences on proximate composition of *L. thoracis* muscles, as Angus had greater EE content vs. Nellore animals. Additionally, it is important to mention that values of intramuscular fat of Nellore cattle in Teixeira et al. (2017) were approximately 3 times greater than those observed herein (4.31 vs. 1.45%). These differences could be related to the inclusion of corn in the diets, given that age of the animals and corn processing might not account for such differences among studies. Nonetheless, EE values reported herein are in agreement to previous data in the literature for *B. indicus* breeds (Andrade et al., 2009). Hence, it is yet to be determined the possible, if any, effects of specific FA on proximate composition of samples obtained from *B. indicus* animals slaughtered under a high-concentrate diet setting.

The modulation of the nutritional profile of edible products, such as milk and meat, is directly affected by the diet offered to the animals. As reported, beef is rich in several nutrients frequently related to cardiovascular diseases due to the proportion of SFA and cholesterol, but increases on C18:1 *cis-9* content would be beneficial to health of meat consumers (Smith and Johnson, 2016). Moreover, the amount of FA and its composition in beef varies among breeds, nutrition, sex, and carcass finishing level of the beef cattle herd (Rule et al., 1997). Hence, it was hypothesized that offering lipid supplements with different FA profiles would result in significant changes in meat FA profile. In agreement with previous studies using Nellore cattle (Correa et al., 2012; Cesar et al., 2014), C18:1 *cis-9* and C16:0 were the major FA in *L. thoracis* steaks, whereas a high n-6:n-3 FA ratio (> 4:1) was observed. This latter fact has been shown to have beneficial effects on health of patients with chronic diseases, such as a 70% decrease in total mortality due to cardiovascular disease (Simopoulos, 2002).

It was speculated that the lipid source and FA profile of the diet would result in different meat FA profile of C14:0 and C16:0, which are known as hypercholesterolemic FA (Farfan, 1996). This was even more expected due to differences observed on intake of these FA. Nonetheless, no treatment differences were observed on meat % of C14:0 and C16:0 FA, which can be attributed, at least for C16:0 FA, to the greater mRNA expression of SCD in CONT and MIXT and the probable conversion of this FA to C18:0 and C18:1 *cis-9.* Additionally, the greater meat % of C18:2 CLA intermediates on CSSO-supplemented cohorts indicate a reduction in rumen pH and a consequent greater degree of ruminal biohydrogenation of C18:2 FA. In agreement with this rationale, Jenkins et al. (2008) demonstrated that C18:2 *cis*-9, *trans*-11 CLA is the most prevalent isomer found in ruminal contents of cattle and C18:1 *trans*-10 is also originated from C18:2 FA.

Marbling is highly affected by diets offered to ruminants, so that ingredients that increase propionate production (i.e., corn) have greater glycogenic and insulinogenic capacities, which would increase intramuscular fat deposition (Gilbert et al., 2003). However, this was not verified in this experiment even though significant treatment differences were observed on starch intake. Nonetheless, it is important to mention that many of the genes that control adipocyte differentiation in cattle is regulated by FA circulating in plasma (Choi et al., 2015). Oleic acid has been recognized as potent in stimulating lipid synthesis from glucose in intramuscular tissue and lipogenesis from acetate (Smith and Johnson, 2016). Hence, due to differences in C16:0, C18:0, C18:1 *cis*-9, and C18:2 FA intakes observed herein, differences on marbling and expression of enzymes involved in lipid metabolism were also expected. Peroxisome proliferator-activated receptor-γ has been a gene of interest when looking at lipid metabolism and its potential effects on meat characteristics, such as marbling (Lim et al., 2011), whereas it has been reported that SFA vs. UFA are more potent inducers of PPAR-γ (Bionaz et al., 2012). However, this may not be always true, as Schubach et al. (2019) reported a greater expression of PPAR-γ mRNA in beef calves offered a PUFA-enriched supplement through creep-feeding from 2 to 4 mo of age. Additionally, PUFA supplementation to newly weaned beef calves until the animals are approximately 8 mo of age has consistently improved carcass marbling at slaughter (Cooke et al., 2011; Mangrum et al., 2016), demonstrating that PUFA might cause metabolic imprinting effects through the upregulation of PPAR-γ and that the potential stimulatory effects of PUFA in this gene might depend on the age of the animal.

Conversely to what was reported on PPAR-γ mRNA, CSSO supplementation reduced mRNA expression of SCD, LPL, and SREBP-1. Moreover, CSVO supplementation also reduced the mRNA expression of SCD vs. CONT cohorts. These results do not agree with Schubach et al. (2019) in which effects were either positive or similar between CSSO- and SFA-supplemented cohorts. Additionally, the reason why the results herein were different is intriguing, given that in agreement to PPAR-γ effects on lipid metabolism, enhanced SREBP-1 expression indicates a greater capacity of de novo FA synthesis and lipid accumulation in muscle tissue (Zhao et al., 2010). Greater expression of SCD has been associated with adipocytes hyperthrophy (Martin et al., 1999) and there has been reported an association between SREBP-1 and SCD (Waters et al., 2009). Waters et al. (2009) demonstrated that fish oil supplementation to beef cattle reduced SCD and SREBP-1 mRNA expression when compared with soybean oil. Conversely, in agreement to our results, a negative correlation was observed between tissue C18:2 *cis-*9*, trans*-11 CLA concentrations and SCD (Keating et al., 2005) and SREBP-1 (Waters et al., 2009) expression. *In vitro* addition of C16:0 and C18:0 increased mRNA expression of SCD, whereas its expression decreased following C18:1 *cis*-9 and C18:2 *cis*-9, *cis*-12 addition (Smith and Johnson, 2016). Moreover, these same authors also reported that supplementation with C16:0 increased marbling, but decreased SCD activity likely due to C18:1 *cis-*9 content in the diet. In the present experiment, as rumen FA biohydrogenation was not measured, it could be speculated that although a greater C18:1 *cis-*9 FA intake was observed in CONT and CSVO, a greater biohydrogenation was also observed, equally stimulating SCD expression in the tissue of ruminants.

Lipoprotein lipase’s main function is to hydrolyze TG-rich lipoproteins, especially chylomicrons and VLDL, thereby generating free FA and glycerol for energy metabolism and storage (Goldberg, 1996). Recently, Teixeira et al. (2017) reported a close association between SCD and LPL tissue expression, so it is not surprising the treatment differences reported herein for mRNA LPL expression. Although previous studies in the literature reported greater mRNA expression of genes involved in lipid metabolism following n-6 FA supplementation, one might speculate that the greater content of C18:2 *cis-*9, *trans*-11 CLA in the meat of *B. indicus* animals inhibited the expression of enzymes involved in lipid metabolism and evaluated herein, such as LPL, SREBP-1c, and SCD.

Meat quality attributes, such as color, tenderness, flavor, and juiciness, have major effects on consumer satisfaction (Dransfield et al., 2003). Additionally, MUFA concentrations in meat have been shown to influence beef palatability, as greater C18:1 *cis-9* FA contents were associated with greater overall palatability (Westerling and Hedrick, 1979). More specifically, meat color is affected by fat deposition and oxidation processes (Fruet et al., 2016) and higher L* (lightness) values are positively correlated to b* (yellowness; Luciano et al., 2009), whereas both are related to total lipids in muscle. According to our results, MIXT supplementation yielded meats with reduced L* and a* values, whereas b* indexes were reduced for CSSO and MIXT vs. CONT. The reason and/or mechanisms by which differences were observed on color parameters are unknown, as treatments did not affect meat pH and colorimetric traits are highly correlated to pH. In agreement with our results, Gilbert et al. (2003) also did not report effects of different lipid sources and FA profile of supplements on cooking loss and WBSF. Overall, L* and pH results observed herein are in agreement to those reported by Sant’Anna et al. (2019) in meat carcass characteristics of Nellore cattle, whereas WBSF, a*, and b* indexes differed considerably.

It is reported in the literature that steaks with WBSF values greater than 4.6 kg/cm^2^ are hard (Belew et al., 2003) and our results suggest that animals, regardless of treatment, had lower WBSF values and, consequently, steaks were softer than preivously reported in the literature for *B. indicus* cattle. In agreement with our results, Oliveira et al. (2012) did not report positive effects of CSFA supplementation on meat WBSF of Nellore animals. These authors reported higher WBSF values (5.97 kg/cm^2^), suggesting that Nellore cattle present more collagen crosslinks and greater calpastatin, which in turn will cause a reduction in calpain activity. Others have also reported that zebu breeds tend to present less tender meat when compared with taurus cohorts (Smith et al., 2009), likely due to a greater mithocondrial efficiency on ATP production that leads to a greater resistance on meat pH drops (Ramos et al., 2020a; Ramos et al., 2020b). Other factors, such as cattle temperament and age, might play a role in consistently and permanently affecting carcass characteristics of *B. indicus* cattle, whereas the specific effects of CSFA, if any, are yet to be determined.

In the present experiment, supplementation with CSSO improved flavor and aroma of steaks when compared with CONT and CSVO, whereas MIXT also had reduced flavor grades vs. CSSO and did not differ on aroma evaluations. Conversely, Gilbert et al. (2003) did not report differences on a meat sensorial panel of steaks obtained from animals offered different lipid sources and FA profile. A general concern of providing PUFA to cattle is a possible development of off-flavors associated with n-6 and n-3 FA and their derivatives in meat or from other components in calcium salts of fatty acids. Overall, C18:1 *cis-*9 leads to a greater flavor acceptance, whereas n-6 has been associated with decreased juiciness and n-3 with decreased flavor acceptance (Melton et al., 1982). Nonetheless, meat obtained from CSSO animals had greater content of CLA intermediates and C18:1 *trans*-10, whereas a reduced C18:0 concentration compared with other treatments.

In summary, the addition of different lipid sources with varying FA profiles into high-concentrate diets did not affect performance and carcass characteristics of *B. indicus* beef animals. Nonetheless, the supplementation with calcium salts of soybean oil downregulated the mRNA expression of enzymes involved in lipid metabolism, whereas flavor and aroma were positively affected by this lipid source. Hence, nutritional alternatives are available that could be employed into feedlot diets to alter characteristics of the meat and provide a better acceptance within the customer chain.

## References

[CIT0001] Abularach M L S, Rocha C E, and Felicio P E. 1998 Características de qualidade do contrafilé (m. *L. dorsi*) de touros jovens da raça Nelore. Food Sci. Technol. 18:205–210. doi:10.1590/S0101-206119980002000012

[CIT0002] Allen M S, Bradford B J, and Oba M. 2009 Board invited review: the hepatic oxidation theory of the control of feed intake and its application to ruminants. J. Anim. Sci. 87:3317–3334. doi:10.2527/jas.2009-1779. 19648500

[CIT0003] AMSA. American Meat Science Association. 1995 Research guidelines for cookery, sensory evaluation, and instrumental tenderness measurements of fresh meat. AMSA & National Livestock and Meat Board, Chicago.

[CIT0004] Andrade P L, Bressan M C, Gama I, Gonçalves T, Ladeira M M, and Ramos E M. 2009 Aged meat quality in Red Norte and Nellore cattle. Rev. Bras. Zootec. 39:1791–1800. doi:10.1590/S1516-359820100000800023

[CIT0005] AOAC 1990 Official methods of analysis. 15th ed. Association of Official Analytical Chemist, Washington, DC.

[CIT0006] AOAC 2007 Official methods of analysis. 18th ed. Association of Official Analytical Chemists, Washington, DC.

[CIT0007] AOCS 2009 Official methods and recommended practices. 7th ed. American Oil Chemists’ Society, Urbana, IL.

[CIT0008] Archibeque S L, Lunt D K, Gilbert C D, Tume R K, and Smith S B. 2005 Fatty acid indices of stearoyl-CoA desaturase do not reflect actual stearoyl-CoA desaturase enzyme activities in adipose tissues of beef steers finished with corn-, flaxseed-, or sorghum-based diets. J. Anim. Sci. 83:1153–1166. doi:10.2527/2005.8351153x. 15827260

[CIT0009] Belew J B, Brooks J C, McKenna D R, and Savell J W. 2003 Warner-Bratzler shear evaluations of 40 bovine muscles. Meat Sci. 64:507–512. doi:10.1016/S0309-1740(02)00242-5. 22063134

[CIT0010] Bevans D W, Beauchemin K A, Schwartzkopf-Genswein K S, McKinnon J J, and McAllister T A. 2005 Effect of rapid or gradual grain adaptation on subacute acidosis and feed intake by feedlot cattle. J. Anim. Sci. 83:1116–1132. doi:10.2527/2005.8351116x. 15827257

[CIT0011] Bionaz M, Thering B J, and Loor J J. 2012 Fine metabolic regulation in ruminants via nutrient-gene interactions: saturated long-chain fatty acids increase expression of genes involved in lipid metabolism and immune response partly through PPAR-α activation. Br. J. Nutr. 107:179–191. doi:10.1017/S0007114511002777. 21729373

[CIT0012] Boekholdt S M, Souverein O W, Tanck M W, Hovingh G K, Kuivenhoven J A, Peters R I, Jansen H, Schiffers P M, van der Wall E E, Doevendans P A, et al. 2006 Common variants of multiple genes that control reverse cholesterol transport together explain only a minor part of the variation of HDL cholesterol levels. Clin. Genet. 69:263–270. doi:10.1111/j.1399-0004.2006.00578.x. 16542392

[CIT0013] Cappellozza B I, Cooke R F, Reis M M, Moriel P, Keisler D H, and Bohnert D W. 2014 Supplementation based on protein or energy ingredients to beef cattle consuming low-quality cool-season forages: II. Performance, reproductive, and metabolic responses of replacement heifers. J. Anim. Sci. 92:2725–2734. doi:10.2527/jas.2013-7442. 24713166

[CIT0014] Cesar A S M, Regitano L C A, Mourão G B, Tullio R R, Lanna D P D, Nassu R T, Mudado M A, Oliveira P S N, Nascimento M L, Chaves A S, et al. 2014 Genome-wide association study for intramuscular fat deposition and composition in Nellore cattle. BMC Genet. 15:39. doi:10.1186/1471-2156-15-39 24666668 PMC4230646

[CIT0080] Choi M S, Kim Y J, Kwon E Y, Ryoo J Y, Kim S R, and Jung U J. 2015 High-fat diets decreases energy expenditure and expression of genes controlling lipid metabolism, mitochondrial function and skeletal system development in the adipose tissue, along with increased expression of extracelular matrix remodelling- and inflammation-related genes. Br. J. Nutr. 113:867–877. doi:10.1017/S0007114515000100. 25744306

[CIT0015] Choi S H, Gang G O, Sawyer J E, Johnson B J, Kim K H, Choi C W, and Smith S B. 2013 Fatty acid biosynthesis and lipogenic enzyme activities in subcutaneous adipose tissue of feedlot steers fed supplementary palm oil or soybean oil. J. Anim. Sci. 91:2091–2098. doi:10.2527/jas.2012-5801. 23463571

[CIT0016] Cooke R F, Bohnert D W, Moriel P, Hess B W, and Mills R R. 2011 Effects of polyunsaturated fatty acid supplementation on ruminal in situ forage degradability, performance, and physiological responses of feeder cattle. J. Anim. Sci. 89:3677–3689. doi:10.2527/jas.2010-3515. 21680784

[CIT0017] Correa L B, Zanetti M A, Del Claro G R, de Melo M P, Rosa A F, and Saran Netto A. 2012 Effect of supplementation of two sources and two levels of copper on lipid metabolism in Nellore beef cattle. Meat Sci. 91:466–471. doi:10.1016/j.meatsci.2012.02.033. 22444665

[CIT0018] de Souza J, Garver J L, Preseault C L, and Lock A L. 2017 Short communication: Effects of prill size of a palmitic acid-enriched fat supplement on the yield of milk and milk components, and nutrient digestibility of dairy cows. J. Dairy Sci. 100:379–384. doi:10.3168/jds.2016-11610. 28341047

[CIT0019] de Souza J, and Lock A L. 2018 Short communication: Comparison of a palmitic acid-enriched triglyceride supplement and calcium salts of palm fatty acids supplement on production responses of dairy cows. J. Dairy Sci. 101:3110–3117. doi:10.3168/jds.2017-13560. 29397168

[CIT0021] Dransfield E, Martin J-F, Bauchart D, Abouelkaram S, Lepetit J, Culioli J, Jurie C, and Picard B. 2003 Meat quality and composition of three muscles from French cull cows and young bulls. Anim. Sci. 76:387–399. doi:10.1017/S1357729800058616

[CIT0023] Farfan J A 1996 Foods that influence cholesterol levels in the body. In: Institute of food technology. Lecture “Cholesterol”: Analysis, occurrence, reduction in foodand health implications. ITAL, Campinas pp. 35–44.

[CIT0024] Fiorentini G, Carvalho I P, Messana J D, Castagnino P S, Berndt A, Canesin R C, Frighetto R T, and Berchielli T T. 2014 Effect of lipid sources with different fatty acid profiles on the intake, performance, and methane emissions of feedlot Nellore steers. J. Anim. Sci. 92:1613–1620. doi:10.2527/jas.2013-6868. 24492580

[CIT0025] Fiorentini G, Messana J D, Dian P H M, Reis R A, Canesin R C, Pires A V, and Berchielli T T. 2013 Digestibility, fermentation, and rumen microbiota in crossbred heifers fed different dietary lipid sources. Anim. Feed Sci. Technol. 181:26–34. doi:10.1016/j.anifeedsci.2013.01.011

[CIT0026] Fleige S, and Pfaffl M W. 2006 RNA integrity and the effect on the real-time qRT-PCR performance. Mol. Aspects Med. 27:126–139. doi:10.1016/j.mam.2005.12.003. 16469371

[CIT0027] Folch J, Lees M, and Sloane Stanley G H. 1957 A simple method for the isolation and purification of total lipides from animal tissues. J. Biol. Chem. 226:497–509. 13428781

[CIT0028] Fox D G, Tedeschi L O, Tylutki T P, Russell J B, Van Amburgh M E, Chase L E, Pell A N, and Overton T R. 2004 The Cornell Net Carbohydrate and Protein System model for evaluating herd nutrition and nutrient excretion. Anim. Feed Sci. Technol. 112:29–78. doi:10.1016/j.anifeedsci.2003.10.006.

[CIT0029] Friedewald W T, Levy R I, and Fredrickson D S. 1972 Estimation of the concentration of low-density lipoprotein cholesterol in plasma, without use of the preparative ultracentrifuge. Clin. Chem. 18:499–502. doi:10.1093/clinchem/18.6.499. 4337382

[CIT0030] Fruet A P, Stefanello F S, Rosado Júnior A G, Souza A N, Tonetto C J, and Nörnberg J L. 2016 Whole grains in the finishing of culled ewes in pasture or feedlot: Performance, carcass characteristics and meat quality. Meat Sci. 113:97–103. doi:10.1016/j.meatsci.2015.11.018. 26638020

[CIT0031] Gilbert C D, Lunt D K, Miller R K, and Smith S B. 2003 Carcass, sensory, and adipose tissue traits of Brangus steers fed casein-formaldehyde-protected starch and/or canola lipid. J. Anim. Sci. 81:2457–2468. doi:10.2527/2003.81102457x. 14552372

[CIT0032] Goldberg I J 1996 Lipoprotein lipase and lipolysis: central roles in lipoprotein metabolism and atherogenesis. J. Lipid Res. 37:693–707. 8732771

[CIT0033] Hales K E, Wells J E, Berry E D, Kalchayanand N, Bono J L, and Kim M. 2017 The effects of monensin in diets fed to finishing beef steers and heifers on growth performance and fecal shedding of Escherichia coli O157:H7. J. Anim. Sci. 95:3738–3744. doi:10.2527/jas.2017.1528. 28805884

[CIT0034] Harvatine K J, and Allen M S. 2006 Effects of fatty acid supplements on feed intake, and feeding and chewing behavior of lactating dairy cows. J. Dairy Sci. 89:1104–1112. doi:10.3168/jds.S0022-0302(06)72178-6. 16507707

[CIT0035] Hess B W, Moss G E, Rule D C. 2008 A decade of developments in the area of fat supplementation research with beef cattle and sheep. J. Anim. Sci. 86(Suppl.):E188–E204. doi:10.2527/jas.2007-0546 18156350

[CIT0036] Hocquette J F, Gondret F, Baéza E, Médale F, Jurie C, and Pethick D W. 2010 Intramuscular fat content in meat-producing animals: development, genetic and nutritional control, and identification of putative markers. Animal. 4:303–319. doi:10.1017/S1751731109991091 22443885

[CIT0037] Honikel K O 1998 Reference methods for the assessment of physical characteristics of meat. Meat Sci. 49:447–457. doi:10.1016/s0309-1740(98)00034-5. 22060626

[CIT0038] Houben J H, van Dijk A, Eikelenboom G, and Hoving-Bolink A H. 2000 Effect of dietary vitamin E supplementation, fat level and packaging on colour stability and lipid oxidation in minced beef. Meat Sci. 55:331–336. doi:10.1016/s0309-1740(99)00161-8. 22061291

[CIT0039] Huntington G B 1997 Starch utilization by ruminants: from basics to the bunk. J. Anim. Sci. 75:852–867. doi:10.2527/1997.753852x. 9078506

[CIT0040] Jenkins T C 1993 Lipid metabolism in the rumen. J. Dairy Sci. 76:3851–3863. doi:10.3168/jds.S0022-0302(93)77727-9. 8132891

[CIT0041] Jenkins T C, Wallace R J, Moate P J, and Mosley E E. 2008 Board-invited review: recent advances in biohydrogenation of unsaturated fatty acids within the rumen microbial ecosystem. J. Anim. Sci. 86:397–412. doi:10.2527/jas.2007-0588. 18042812

[CIT0042] Keating A F, Stanton C, Murphy J J, Smith T J, Ross R P, and Cairns M T. 2005 Isolation and characterization of the bovine Stearoyl-CoAdesaturase promoter and analysis of polymorphisms in the promoter region in dairy cows. Mamm. Genome 16:184–193. doi:10.1007/s00335-004-2325-0. 15834635

[CIT0043] Kramer J K, Fellner V, Dugan M E, Sauer F D, Mossoba M M, and Yurawecz M P. 1997 Evaluating acid and base catalysts in the methylation of milk and rumen fatty acids with special emphasis on conjugated dienes and total trans fatty acids. Lipids 32:1219–1228. doi:10.1007/s11745-997-0156-3. 9397408

[CIT0044] Liepa G U, Beitz D C, and Linder J R. 1978 Cholesterol synthesis in ruminating and nonruminating goats. J. Nutr. 108:535–543. doi:10.1093/jn/108.3.535. 627924

[CIT0045] Lim D, Kim N K, Park H S, Lee S H, Cho Y M, Oh S J, Kim T H, and Kim H. 2011 Identification of candidate genes related to bovine marbling using protein-protein interaction networks. Int. J. Biol. Sci. 7:992–1002. doi:10.7150/ijbs.7.992. 21912507 PMC3164149

[CIT0046] Livak K J, and Schmittgen T D. 2001 Analysis of relative gene expression data using real-time quantitative PCR and the 2(-Delta Delta C(T)) Method. Methods 25:402–408. doi:10.1006/meth.2001.1262. 11846609

[CIT0047] Luciano G, Monahan F J, Vasta V, Pennisi P, Bella M, and Priolo A. 2009 Lipid and colour stability of meat from lambs fed fresh herbage or concentrate. Meat Sci. 82:193–199. doi:10.1016/j.meatsci.2009.01.010. 20416762

[CIT0048] Mangrum K S, Tuttle G, Duckett S K, Sell G S, Krehbiel C R, and Long N M. 2016 The effect of supplementing rumen undegradable unsaturated fatty acids on marbling in early-weaned steers. J. Anim. Sci. 94:833–844. doi:10.2527/jas.2015-9809. 27065154

[CIT0049] Marques R S, Bohnert D W, de Sousa O A, Brandão A P, Schumaher T F, Schubach K M, Vilela M P, Rett B, and Cooke R F. 2019 Impact of 24-h feed, water, or feed and water deprivation on feed intake, metabolic, and inflammatory responses in beef heifers. J. Anim. Sci. 97:398–406. doi:10.1093/jas/sky397. 30312410 PMC6313126

[CIT0050] Marques R S, Cooke R F, Francisco C L, and Bohnert D W. 2012 Effects of twenty-four hour transport or twenty-four hour feed and water deprivation on physiologic and performance responses of feeder cattle. J. Anim. Sci. 90:5040–5046. doi:10.2527/jas.2012-5425. 22851237

[CIT0051] Martin G S, Lunt D K, Britain K G, and Smith S B. 1999 Postnatal development of stearoyl coenzyme A desaturase gene expression and adiposity in bovine subcutaneous adipose tissue. J. Anim. Sci. 77:630–636. doi:10.2527/1999.773630x. 10229358

[CIT0052] Mashek D G, and Grummer R R. 2003 Effects of long chain fatty acids on lipid and glucose metabolism in monolayer cultures of bovine hepatocytes. J. Dairy Sci. 86:2390–2396. doi:10.3168/jds.S0022-0302(03)73833-8. 12906057

[CIT0053] Meilgaard M, Civille G V, and Carr B T. 1999 Sensory evaluation techniques. CRC Press, Boca Raton, FL.

[CIT0054] Melton S L, Amiri M, Davis G W, and Backus W R. 1982 Flavor and chemical characteristics of ground beef from grass-, forage-grain- and grain-finished steers. J. Anim. Sci. 55:77–87. doi:10.2527/jas1982.55177x

[CIT0055] Moore C B, Bass P D, Green M D, Chapman P L, O’Connor M E, Yates L D, Scanga J A, Tatum J D, Smith G C, and Belk K E. 2010 Establishing an appropriate mode of comparison for measuring the performance of marbling score output from video image analysis beef carcass grading systems. J. Anim. Sci. 88:2464–2475. doi:10.2527/jas.2009-2593. 20348376

[CIT0056] Mustad V A, Etherton T D, Cooper A D, Mastro A M, Pearson T A, Jonnalagadda S S, and Kris-Etherton P M. 1997 Reducing saturated fat intake is associated with increased levels of LDL receptors on mononuclear cells in healthy men and women. J. Lipid Res. 38:459–468. 9101427

[CIT0057] NASEM 2016 National Academies of Sciences, Engineering, and Medicine. Nutrient requirements of beef cattle model. 8th rev. ed. National Academic Press, Washington, DC.

[CIT0081] Nascimento F A 2017 Supplementation of rumen-protected fat with different fatty acid profiles to feedlot beef cattle [MSc thesis]. Jaboticabal (Brazil): Univ. Estad. Paulista (UNESP).

[CIT0058] Noro A, and Kobayashi Y. 1995 The relationship between serum lipoprotein levels and marbling of muscle tissue in beef cattle. J. Vet. Med. Sci. 57:737–738. doi:10.1292/jvms.57.737. 8519908

[CIT0059] Oliveira E A, Sampaio A A, Henrique W, Pivaro T M, Rosa B L, Fernandes A R, and Andrade A T. 2012 Quality traits and lipid composition of meat from Nellore young bulls fed with different oils either protected or unprotected from rumen degradation. Meat Sci. 90:28–35. doi:10.1016/j.meatsci.2011.05.024. 21680103

[CIT0060] Pinto A C J, and Millen D D. 2016 Current situation of finishing feedlot cattle and nutritional models used. *In* Sebastião de Campos Valadares Filho et al. (Org). Beef Cattle Production Symposium (X Simcorte). 1st ed. Viçosa/MG. 1:103–120.

[CIT0061] Ramos P M, Li C, Elzo M A, Wohlgemuth S E, and Scheffler T L. 2020a Mitochondrial oxygen consumption in early *postmortem* permeabilized skeletal muscle fibers is influenced by cattle breed. J. Anim. Sci. 98:1–10. doi:10.1093/jas/skaa044 10.1093/jas/skaa044 PMC7071943 32171017

[CIT0062] Ramos P M, Wright S A, Delgado E F, van Santen E, Johnson D D, Scheffler J M, Elzo M A, Carr C C, and Scheffler T L. 2020b Resistance to pH decline and slower calpain-1 autolysis are associated with higher energy availability early *postmortem* in *Bos taurus indicus* cattle. Meat Sci. 159:107925. doi:10.1016/j.meatsci.2019.107925. 31476681

[CIT0063] Rule D C, MacNeil M D, and Short R E. 1997 Influence of sire growth potential, time on feed, and growing-finishing strategy on cholesterol and fatty acids of the ground carcass and longissimus muscle of beef steers. J. Anim. Sci. 75:1525–1533. doi:10.2527/1997.7561525x. 9250513

[CIT0064] Samuelson K L, Hubbert M E, and Löest C A. 2016 Effects of dietary urea concentration and zilpaterol hydrochloride on performance and carcass characteristics of finishing steers. J. Anim. Sci. 94:5350–5358. doi:10.2527/jas.2016-0875. 28046136

[CIT0082] Sant’Anna A C, Valente T S, Magalhães A F B, Espigolan R, Ceballos M C, Albuquerque L G, and Paranhos da Costa M J R. 2019 Relationships between temperament, meat quality, and carcass traits in Nellore cattle. J. Anim. Sci. 97:4721–4737. doi:10.1093/jas/skz324. 31616922 PMC6915207

[CIT0065] Scholljegerdes E J, Lake S L, Weston T R, Rule D C, Moss G E, Nett T M, and Hess B W. 2007 Fatty acid composition of plasma, medial basal hypothalamus, and uterine tissue in primiparous beef cows fed high-linoleate safflower seeds. J. Anim. Sci. 85:1555–1564. doi:10.2527/jas.2005-732. 17325123

[CIT0066] Schubach K M, Cooke R F, Brandão A P, de Sousa O A, Schumaher T F, Jump D B, Pohler K G, Bohnert D W, and Marques R S. 2019 Supplementing calcium salts of soybean oil to beef steers early in life to enhance carcass development and quality1. J. Anim. Sci. 97:4182–4192. doi:10.1093/jas/skz272. 31425585 PMC6776389

[CIT0067] Simopoulos A P 2002 The importance of the ratio of omega-6/omega-3 essential fatty acids. Biomed. Pharmacother. 56:365–379. doi:10.1016/s0753-3322(02)00253-6. 12442909

[CIT0083] Smith S B, and Johnson B J. 2016 Marbling: management of cattle to maximize the depositionof intramuscular adipose tissue. J. Anim. Sci. 94(Suppl. 5):382. doi:10.2527/jam2016-0794.

[CIT0068] Smith S B, Lunt D K, Chung K Y, Choi C B, Tume R K, and Zembayashi M. 2006 Adiposity, fatty acid composition, and delta-9 dessaturase activity during growth in beef cattle. Anim. Sci. J. 77:478–486. doi:10.1111/j.1740-0929.2006.00375.x

[CIT0069] Smith T, Thomas M G, Bidner T D, Paschal J C, and Franke D E. 2009 Single nucleotide polymorphisms in Brahman steers and their association with carcass and tenderness traits. Genet. Mol. Res. 8:39–46. doi:10.4238/vol8-1gmr537. 19224465

[CIT0070] Sukhija P S, and Palmquist D L. 1990 Dissociation of calcium soaps of long-chain fatty acids in rumen fluid. J. Dairy Sci. 73:1784–1787. doi:10.3168/jds.S0022-0302(90)78858-3. 2229592

[CIT0071] Teixeira P D, Oliveira D M, Chizzotti M L, Chalfun-Junior A, Coelho T C, Gionbelli M, Paiva L V, Carvalho J R R, and Ladeira M M. 2017 Subspecies and diet affect the expression of genes involved in lipid metabolism and chemical composition of muscle in beef cattle. Meat Sci. 133:110–118. doi:10.1016/j.meatsci.2017.06.009. 28666109

[CIT0072] UGC. Ultrasound Guidelines Council. 2014 Field technician study guide. [accessed May 12, 2020]. Available from http://www.ultrasoundbeef.com/uploads/Study_Guide_Chapters_-_2012.zip.

[CIT0073] Van Soest P J, Robertson J B, and Lewis B A. 1991 Methods for dietary fiber, neutral detergent fiber, and nonstarch polysaccharides in relation to animal nutrition. J. Dairy Sci. 74:3583–3597. doi:10.3168/jds.S0022-0302(91)78551-2. 1660498

[CIT0074] Waters S M, Kelly J P, O’Boyle P, Moloney A P, and Kenny D A. 2009 Effect of level and duration of dietary n-3 polyunsaturated fatty acid supplementation on the transcriptional regulation of Delta9-desaturase in muscle of beef cattle. J. Anim. Sci. 87:244–252. doi:10.2527/jas.2008-1005. 18791145

[CIT0075] Weiss W P, Conrad H R, and St. Pierre N R. 1992 A theoretically-based model for predicting total digestible nutrient values of forages and concentrates. Anim. Feed Sci. Technol. 39:95–110. doi:10.1016/0377-8401(92)90034-4

[CIT0076] Westerling D B, and Hedrick H B. 1979 Fatty acid composition of bovine lipids as influenced by diet, sex and anatomical location and relationship to sensory characteristics. J. Anim. Sci. 48:1343–1348. doi:10.2527/jas1979.4861343x

[CIT0077] Wheeler T L, Shackelford S D, and Koohmaraire M. 1997 Standardizing collection and interpretation of Warner-Bratzler shear force and sensory tenderness data. Proceedings of the Reciprocal Meat Conference, Savoy, v. 50, p. 68–77.

[CIT0078] Williams G L, and Stanko R L. 2000 Dietary fats as reproductive nutraceuticals in beef cattle. J. Anim. Sci. 77:1–12. doi:10.2527/jas2000.77E-Suppl1n

[CIT0079] Zhao Y M, Basu U, Dodson M V, Basarb J A, and Guan L L. 2010 Proteome differences associated with fat accumulation in bovine subcutaneous adipose tissues. Proteome Sci. 8:14. doi:10.1186/1477-5956-8-14. 20298566 PMC2853513

